# Secondary structure and ^1^H, ^15^ N & ^13^C resonance assignments of the periplasmic domain of OutG, major pseudopilin from *Dickeya dadantii* type II secretion system

**DOI:** 10.1007/s12104-022-10085-4

**Published:** 2022-04-28

**Authors:** Theis Jacobsen, Régine Dazzoni, Melvin G. Renault, Benjamin Bardiaux, Michael Nilges, Vladimir Shevchik, Nadia Izadi-Pruneyre

**Affiliations:** 1 CNRS UMR3528, Structural Bioinformatics Unit, Institut Pasteur, Université Paris Cité, 75015 Paris, France; 2 Université Claude Bernard Lyon 1, INSA-Lyon, CNRS, UMR5240 MAP, Microbiologie Adaptation et Pathogénie, 69622 Villeurbanne, France; 3 Sorbonne Université, Complexité du Vivant, 75005 Paris, France

**Keywords:** NMR resonance assignments, Type II secretion system, OutG, *Dickeya dadantii*, Pseudopilin

## Abstract

The ability to interact and adapt to the surrounding environment is vital for bacteria that colonise various niches and organisms. One strategy developed by Gram-negative bacteria is to secrete exoprotein substrates via the type II secretion system (T2SS). The T2SS is a proteinaceous complex spanning the bacterial envelope that translocates folded proteins such as toxins and enzymes from the periplasm to the extracellular milieu. In the T2SS, a cytoplasmic ATPase elongates in the periplasm the pseudopilus, a non-covalent polymer composed of protein subunits named pseudopilins, and anchored in the inner membrane by a transmembrane helix. The pseudopilus polymerisation is coupled to the secretion of substrates. The T2SS of *Dickeya dadantii* secretes more than 15 substrates, essentially plant cell wall degrading enzymes. In *D. dadantii*, the major pseudopilin or the major subunit of the pseudopilus is called OutG. To better understand the mechanism of secretion of these numerous substrates via the pseudopilus, we have been studying the structure of OutG by NMR. Here, as the first part of this study, we report the ^1^H, ^15^N and ^13^C backbone and sidechain chemical shift assignment of the periplasmic domain of OutG and its NMR derived secondary structure.

## Biological context

The type II secretion system (T2SS) is a molecular machinery which is widely used by Gram-negative bacteria to specifically secret exoprotein substrates (Korotkov et al. 2012; Thomassin et al. 2017; Gu et al. 2017). The substrates are species-specific and are often enzymes degrading biopolymers of carbohydrates, proteins, lipids and nucleotides (Cianciotto and White 2017). In addition, T2SSs promote secretion of toxins, adhesins or cytochromes that are involved in respiration, motility or biofilm formation and remain attached to the bacterial cells (Nivaskumar and Francetic 2014). The T2SS machinery is made up of twelve to fifteen components referred to as A to O, and spans both the inner and outer membrane. The T2SS could be divided into three functional blocks, the outer membrane pore composed of fifteen copies of the secretin D, the assembly platform comprising the inner membrane proteins F, L, M, C and the associated cytoplasmic ATPase E, which actively assembles a non-covalent polymer of protein subunits, called the pseudopilus, in the periplasm. The pseudopilus is composed of two types of proteins referred to as pseudopilins: the major pseudopilin G composing the bulk of the pseudopilus, and the minor pseudopilins H, I, J and K that form a complex initiating the pseudopilus formation (Nivaskumar and Francetic 2014; Escobar et al. 2021). The minor pseudopilins are present in much lower abundance compared to the major pseudopilin but are crucial for the formation and stability of the pseudopilus and for secretion of substrates. Substrates are basically exported by the Sec or Tat translocator, then folded in the periplasm where they are recruited by T2SS; with the elongation of the pseudopilus they reach the extracellular space through a secretin pore in the outer membrane (Korotkov et al. 2012; Thomassin et al. 2017; Gu et al. 2017). It remains largely unknown how the growing pseudopilus can translocate the T2SS substrates and whether and how substrates are specifically recognized by pseudopilus.

*Dickeya dadantii* is a phytopathogenic γ-proteobacterium, which causes soft rot in vegetables and growing plants (Hugouvieux‐Cotte‐Pattat et al*.* 2020). *D. dadantii* secrete more than 15 substrates via the T2SS (Kazemi-Pour et al. 2004). Most of the characterized substrates are hydrolytic enzymes that degrade the cell wall of plants and thereby release nutrients that the bacteria can take up and metabolise (Hugouvieux-Cotte-Pattat et al. 2014). Occurrence of multiple, structurally dissimilar substrates makes *D. dadantii* an attractive model to study the mechanisms of T2SS.

In this study, we focused on the major pseudopilin from *D. dadantii,* named OutG, as an essential T2SS element. OutG is a 13.5 kDa protein composed of 153 residues. It has a short N-terminal prepilin signal sequence which allows correct insertion in the inner membrane, and is then cleaved by a prepilin peptidase. The mature protein OutG of 146 residues long is anchored in the inner membrane with an N-terminal, 24 residues long, hydrophobic transmembrane helix followed by a C-terminal globular domain of 122 residues located in the periplasm (hereafter termed to as OutGp).

Structural insight into OutG is of high importance for the understanding of the putative interactions of the pseudopilus with the substrates and other T2SS components. Here we present the resonance assignment and derived secondary structure of OutGp as a starting point for its structural and interaction studies by NMR.

## Method and experiments

### Expression and purification of isotope labelled OutGp

The pET20b(+) vector *(Novagen*) was used for the expression of OutGp in the *Escherichia coli* periplasm. The N-terminus of OutGp was successively fused to the PelB signal peptide, followed by a 6His affinity tag and a TEV cleavage site. The hybrid OutGp is exported by Sec translocon into the periplasm and the PelB signal peptide is cleaved off by the LepB signal peptidase. Thereafter, the 6His tag was removed by TEV protease treatment during purification. In this way, the final OutGp protein used in this study is composed of an N-terminal GMG sequence followed by residues M25 to P146 of the mature OutG (122 residues).

Uniformly ^15^N–^13^C double-labelled OutGp was produced in M9 minimal media using 1 g/L of ^15^NH_4_Cl and 4 g/L ^13^C-glucose as the only nitrogen and carbon source, respectively. Protein production was induced by addition of 1 mM IPTG overnight at 18 °C in *E. coli* BL21 (DE3) cells. After induction, the cells were lysed by sonication in equilibration buffer (50 mM Tris–HCl pH 8, 100 mM NaCl, 10 mM Imidazole), then the polynucleotides were digested with nuclease (*Benzonase®, Sigma*) and the cell debris were pelleted by centrifugation at 16,000 g for one hour at 4 °C. The supernatant was loaded onto a HiTrap HP column (*Cytiva*) previously loaded with Ni^2+^ ions, by saturating the resin with 0.1 M NiSO_4_ solution, then equilibrated in equilibration buffer. After loading of the lysate, the column was washed with the equilibration buffer, and bound proteins were eluted with a linear gradient of imidazole from 10 to 300 mM. The eluted protein fractions were pooled and treated with TEV protease overnight at 14 °C, for the cleavage of the N-terminal His-tag. The mixture was loaded onto a HiTrap HP column (*Cytiva)* and the flow-through containing OutGp without its N-terminal His-tag was concentrated in a Vivaspin® 20 (*Satorius*) concentrator with a 5 kDa molecular weight cut-off. The concentrated OutGp fraction was loaded onto a Sephacryl S-100 column (*Cytiva*) equilibrated in 50 mM HEPES, pH 7, 100 mM NaCl, 5 mM CaCl_2_. After elution, the fractions containing OutGp were pooled and concentrated to 0.43 mM for NMR data acquisition. The pH was adjusted to pH 6 by adding 2 μL of HCl at 0.1 M. All steps of the purification were evaluated by SDS-PAGE and in the final NMR sample no contaminants were visible in the final protein preparation subjected to NMR. All buffers used during the purification were supplemented with EDTA-free Protease inhibitor cocktail (*Roche*).

### NMR spectroscopy

All NMR experiments were recorded on a 600 MHz Avance III HD (*Bruker Biospin*) or a 800 MHz Avance NEO (*Bruker Biospin*) spectrometer, both equipped with a cryogenically cooled triple resonance ^1^H [^13^C/^15^N] probe. A standard set of experiments for the ^1^H, ^15^N and ^13^C for backbone and side chain resonance assignment were recorded at 25 °C using NMR experiments implemented in TopSpin 3.6.1 and TopSpin 4.07 (*Bruker Biospin*) for the 600 MHz and 800 MHz, respectively, and IBS libraries (Favier and Brutscher 2019): 2D ^1^H–^15^ N HSQC, ^1^H–^13^C HSQC, HBCBCGCDHD, HBCBCGCDCEHE and 3D HNCA, HN(CO)CA, HNCACB, CBCA(CO)HN, HNCO, HN(CA)CO, HCCH-TOCSY, C(CO)NH-TOCSY, H(CCO)NH-TOCSY, ^15^N-NOESY and ^13^C-NOESY. All proton chemical shifts were referenced to 2,2-dimethyl-2-silapentane-5-sulfonate (DSS) as 0 ppm. ^15^N and ^13^C chemical shifts were referenced indirectly to DSS (Wishart et al. 1995). The data were analysed as previously described (Lopez-Castilla et al. 2017) using CcpNMR Analysis (Vranken et al. 2005), and the prediction of the secondary structure was achieved by analysing the chemical shifts of HN, Hα, Cα, Cβ, C’, and N in TALOS-N (Shen and Bax 2013).

### Extent of assignment and data deposition

**Fig. 1:**
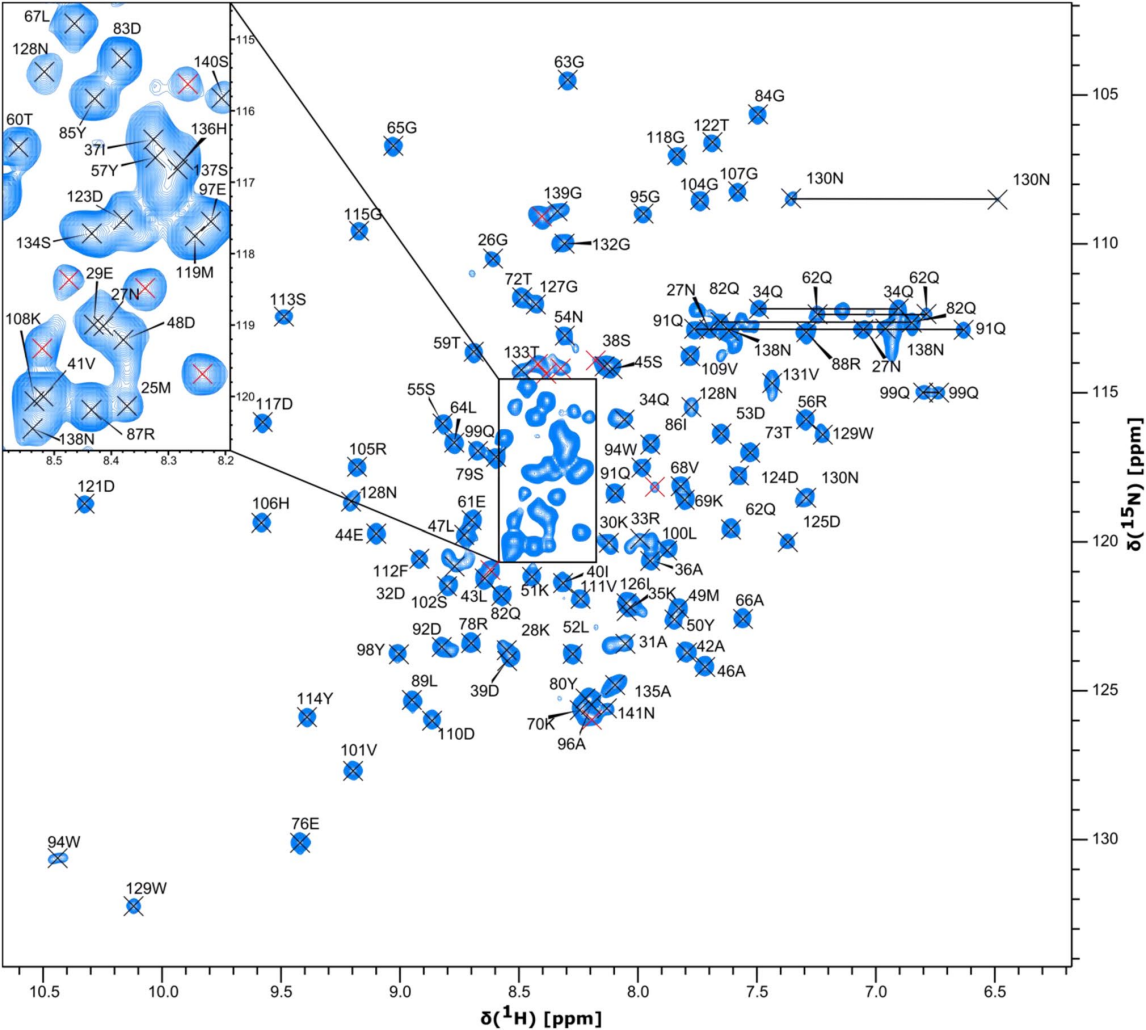
^1^H-^15^N HSQC spectrum of OutGp, major pseudopilin of *Dickeya dadantii*, recorded on a sample of 0.43 mM protein in 50 mM HEPES pH 6, 100 mM NaCl, 5 mM CaCl_2_, 5% D_2_O (v/v), at 25 °C on a 600 MHz Avance III HD (*Bruker Biospin*) spectrometer. The resonance assignment for the backbone amide peaks are displayed using sequence number and one letter amino acid code. The red crosses indicate backbone amide peaks which could not be assigned and corresponding to the region G_142_NGNP_146_. NH_2_ peaks of Asn and Gln sidechains are connected by horizontal lines

High quality data were obtained for OutGp as shown in the ^1^H–^15^N HSQC spectrum in Fig. 1. Backbone amide peaks were observed for all non-proline residues, except for the first three N-terminal residues (G22-G24). In total 96% of the observable backbone resonances (96% of ^1^HN and non-proline ^15^N, 95% of ^13^Cα, 96% of ^13^Cβ, 95% of ^13^CO) and more than 91% of their corresponding sidechain resonances were assigned. Multiple backbone amide peaks of the C-terminal part of OutGp (N_138_GSNGNGNP_146_) were observed indicating that these residues have multiple conformations under the experimental conditions. Consequently, only one conformation of residues N138-N141 from this region could be assigned. It was not possible to assign any resonances corresponding to the last five residues (G_142_NGNP_146_), due to the repetitive nature of the sequence and the presence of a proline residue. The backbone amide peaks that could not be assigned are marked with red crosses in Fig. 1, where all other observable residues are assigned (M25-N141). The chemical shift values have been deposited in the BioMagResBank (http://www.bmrb.wisc.edu/) under the accession number 51296.

### Secondary structure

**Fig. 2:**
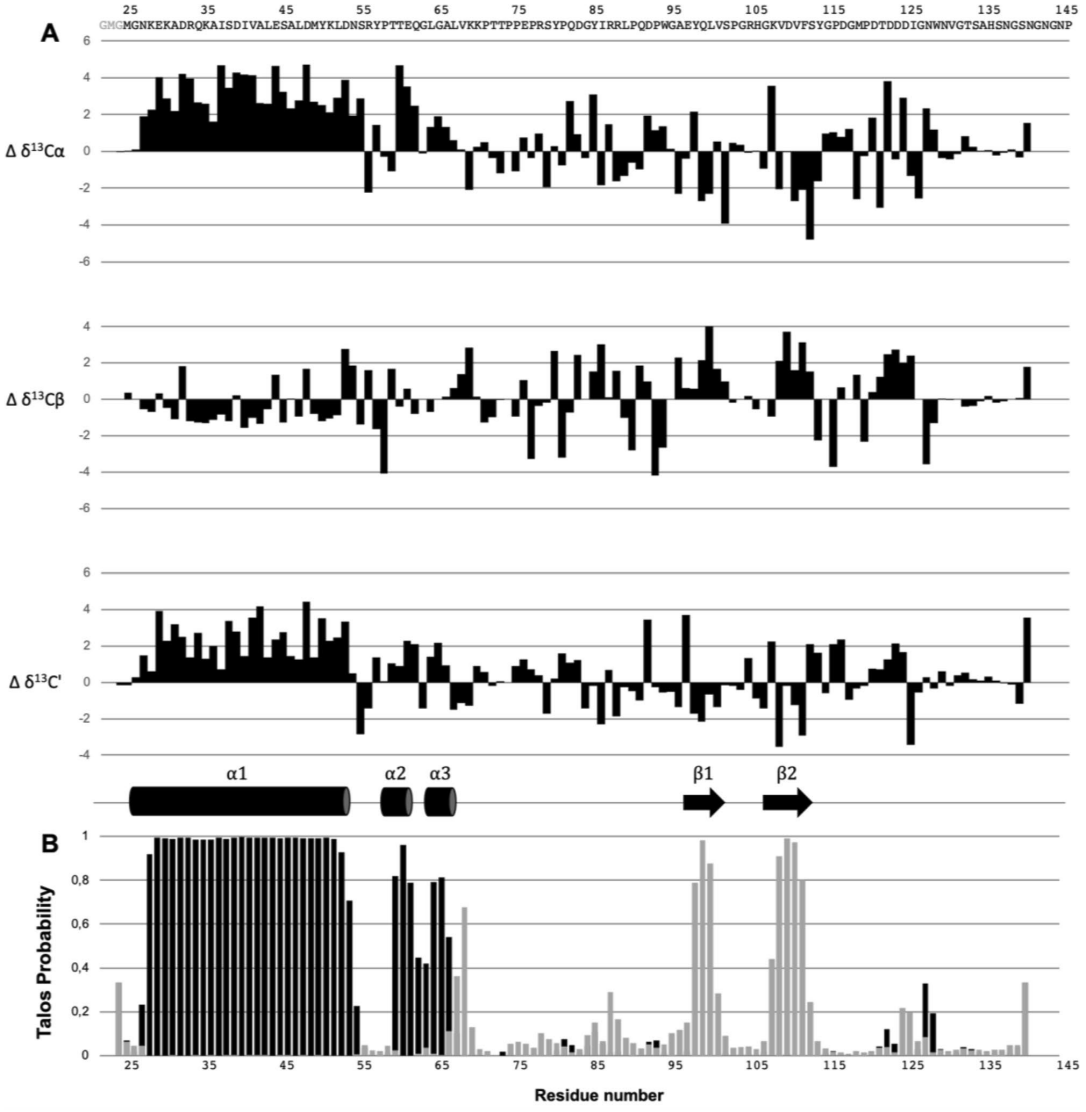
Secondary structure prediction based on the chemical shifts of OutGp. **A** Secondary chemical shifts of C$\alpha$, C$\beta$ and C’ resonances. The sequence of OutGp is shown at the top. **B** TALOS-N secondary structure probabilities were used to predict the secondary structure elements along the protein sequence. The probability for each residue to be in an $\alpha$-helical conformation (black bars) or in $\beta$-sheet conformation (grey bars) are plotted as a function of residue number. Both the secondary chemical shifts and TALOS-N secondary structure prediction agree to a consensus of secondary structure elements illustrated between panel A and B (cylinders represent $\alpha$-helices and arrows represent $\beta$-sheets)

The secondary structure of OutGp was estimated by using two approaches, secondary chemical shifts analysis and TALOS-N prediction (Fig. 2A). Secondary chemical shifts are obtained by calculating the differences between the assigned chemical shifts and the theoretical random coil chemical shift. The value of these secondary chemical shifts for C$\alpha$, C$\beta$ and C’ ($\Delta \delta$^13^C$\alpha$, $\Delta \delta$^13^C$\beta$ & $\Delta \delta$^13^C’) is related to the secondary structure of the individual residues. Positive values of $\Delta \delta$^13^C$\alpha$ and $\Delta \delta$^13^C’ correspond to a $\alpha$-helical conformation whereas negative values correspond to $\beta$-strand structure. For $\Delta \delta$^13^C $\beta$ positive values reflect $\beta$-strand structure and negative values $\alpha$-helical conformation (Fig. 2A).

To complete this prediction, a TALOS-N analysis of the assigned HN, H$\alpha$, C$\alpha$, C $\beta$, C’ and N chemical shifts was performed (Shen and Bax 2013). The TALOS-N probabilities for each residue to be in an $\alpha$-helical conformation or a $\beta$-strand structure is plotted in Fig. 2B.

The results of both analysis methods agree to the location of the secondary structure elements, and reveal three helical segments ($\alpha$1: K28-N54, $\alpha$2: T60-Q62 and $\alpha$3: G65-L67) and two $\beta$-strands ($\beta$1: Q99-V101 and $\beta$2: D110-S113), in the $\alpha$1-$\alpha$2-$\alpha$3-$\beta$1-$\beta$2 sequential order. Structural determination of OutGp will be performed following this study.

## Data Availability

The chemical shift values have been deposited in the BioMagResBank (http://www.bmrb.wisc.edu/) under the accession number 51296.
